# A New Parameter for Calcium Oxalate Stones: Impact of Linear Calculus Density on Non-Contrast Computed Tomography

**DOI:** 10.3390/medicina59020267

**Published:** 2023-01-30

**Authors:** Jae Yong Jeong, Kang Su Cho, Dae Ho Kim, Dae Young Jun, Young Joon Moon, Joo Yong Lee

**Affiliations:** 1 Department of Urology, Severance Hospital, Urological Science Institute, Yonsei University College of Medicine, Seoul 03722, Republic of Korea; 2 Department of Urology, Prostate Cancer Center, Gangnam Severance Hospital, Urological Science Institute, Yonsei University College of Medicine, Seoul 06273, Republic of Korea; 3 Center of Evidence-Based Medicine, Institute of Convergence Science, Yonsei University, Seoul 03722, Republic of Korea

**Keywords:** calcium oxalate, chemistry, tomography, X-ray computed, urolithiasis

## Abstract

*Background and Objectives*: Non-contrast computed tomography (NCCT) is widely used to evaluate urolithiasis. The NCCT attenuation, measured in Hounsfield units (HU), has been evaluated to predict stone characteristics. We propose a novel parameter, linear calculus density (LCD), and analyze variables from NCCT imaging to predict calcium oxalate (CaOx) stones, which are common and challenging to fragment. *Materials and Methods*: We retrospectively reviewed the medical records of patients with urolithiasis between 2014 and 2017. Among those, 790 patients were included. Based on the NCCT pre-treatment, the maximal stone length (MSL), mean stone density (MSD), and stone heterogeneity index (SHI) were obtained. In addition, the variation coefficient of stone density (VCSD = SHI/MSD × 100) and linear calculus density (LCD = VCSD/MSL) were calculated. In accordance with the stone analysis, the patients were divided into two groups (CaOx and non-CaOx groups). The logistic regression model and receiver operating characteristic (ROC) curve were used for predictive modeling. *Results*: In the CaOx group, the SHI, VCSD, and LCD were more significant than in the non-CaOx group (all *p* < 0.001). SHI (OR 1.002, 95% CI 1.001–1.004, *p* < 0.001), VCSD (OR 1.028, 95% CI 1.016–1.041, *p* < 0.001), and LCD (OR 1.352, 95% CI 1.270–1.444, *p* < 0.001) were significant independent factors for CaOx stones in the logistic regression models. The areas under the ROC curve for predicting CaOx stones were 0.586 for SHI, 0.66 for VCSD, and 0.739 for LCD, with a cut-point of 2.25. *Conclusions*: LCD can be a useful new parameter to provide additional information to help discriminate CaOx stones before treatment.

## 1. Introduction

The prevalence of the urolithiasis-related disease is 7% to 13% in North America and 1% to 5% in Asia, with a lifetime-estimated recurrence risk of over 50% [1]. Various treatment modalities are available, ranging from medical to invasive management [2,3]. The size and location of the stones and the composition of the urolithiasis are essential; for example, the cystine stone is resistant to shock wave lithotripsy (SWL), and uric acid stones can be resolved using urine alkalinization [4]. For patients with recurrent stones, analyses of stone compositions are especially important, as they may reveal an underlying metabolic abnormality, including cystinuria. Although a detailed analysis can be performed after the stone has been extracted through Fourier transform infrared (FTIR) spectroscopy [5], several theories have been proposed to predict the composition preoperatively through clinical data, including stone characteristics and urine parameters.

Due to its safety, high sensitivity and specificity, non-contrast computed tomography (NCCT) of the abdomen and pelvis has become standard for diagnosing and evaluating urinary calculi [6]. In addition to providing the location and size of the stone, the NCCT image presents an attenuation index measured in Hounsfield units (HU). Some groups have evaluated the attenuation parameters, including mean stone density (MSD), stone heterogeneity index (SHI), and variation coefficient of stone density (VCSD), for utilization as a pre-treatment predictor of stone composition [7,8,9,10,11]. To our knowledge, however, those parameters have not been assessed to predict calcium oxalate (CaOx) stones, which are the most frequent and are relatively hard to fragment. In addition to the previously mentioned parameters, we suggest the linear calculus density (LCD) be calculated as VCSD per stone length and investigate whether it may be a novel predictor for CaOx stones.

## 2. Materials and Methods

### 2.1. Patient Population

The medical records of 917 patients who had operations or procedures for urolithiasis or whose stones passed spontaneously between December 2014 and February 2017 at a single medical institute were reviewed retrospectively. The operations included not only endoluminal surgery, such as retrograde intrarenal surgery or percutaneous nephrolithotomy, but also laparoscopic or robot-assisted lithotomy. Among them, 790 patients who had both NCCT and stone analyses by FTIR spectroscopy were assigned to this study. Patient age, sex, and pre-treatment urine pH were also ascertained, and the stone composition data was stratified following the Mayo Clinic classification [12]. The stones were categorized into five groups: (1) struvite group (stones containing any amount of struvite); (2) cystine group (stones containing any amount of cystine); (3) uric acid group (stones containing any amount of uric acid); (4) calcium phosphate (CaP) group (stones containing >50% carbonate apatite and stones containing any amount of brushite); and (5) calcium oxalate (CaOx) group (stones containing >50% CaOx with or without any hydroxyapatite). This study protocol was reviewed and approved by the Institutional Review Board of Severance Hospital, Yonsei University Health System, Seoul, Korea (Approval No. 4-2022-0987). However, the requirement for written informed consent of subjects was waived since all patient records and data were anonymized in advance, and the design of this study was retrospective.

### 2.2. NCCT Factors and Stone Analyses

Based on pre-treatment NCCT using the GE Centricity system (GE Healthcare Bio-Sciences Corp., Piscataway, NJ, USA), the maximal stone length (MSL, mm), MSD, average HU of stone, and SHI (the standard deviation of stone HU), of each stone were determined. While measuring the MSL, the HU was determined by localizing the largest elliptical dimension of the stone as the region of interest under the bone window setting. MSD was defined as the mean of HU in the region of interest, SHI being the standard deviation of HU for the same region of interest. In addition, the variation coefficients of stone density (VCSD = SHI/MSD × 100) and LCD, defined as VCSD divided by MSL, were obtained. Stones were sent to GC Laboratories, Yongin, Korea, for quantitative analysis of stone composition through FTIR spectroscopy.

### 2.3. Statistical Analyses

First, the entire patient cohort was classified according to stone composition: struvite, cystine, uric acid, CaOx, and CaP. Second, patients were divided into two groups (CaOx and non-CaOx) and analyzed. Data are presented as mean ± standard deviation unless otherwise indicated. The student’s or Welch’s two-sample *t*-test or the Wilcoxon rank-sum test was used for statistical comparisons of continuous demographic variables. Pearson’s chi-squared test was used to compare categorical variables. Univariate and multivariate analyses were performed to identify the factors related to the CaOx group. Optimal cut-points were generated for significant factors through ROC curves, applying the Youden methodology. For all statistical tests, *p* < 0.05 was considered significant.

All computations were performed using R version 4.2.1 (R Foundation for Statistical Computing, Vienna, Austria; http://www.r-project.org (accessed on 9 December 2022)). The OptimalCutpoints package was used to determine optimal cut-points, sensitivity, specificity, positive predictive value, and negative predictive value.

## 3. Results

### 3.1. Demographic Data According to the Mayo Clinic Classification

**Figure 1:**
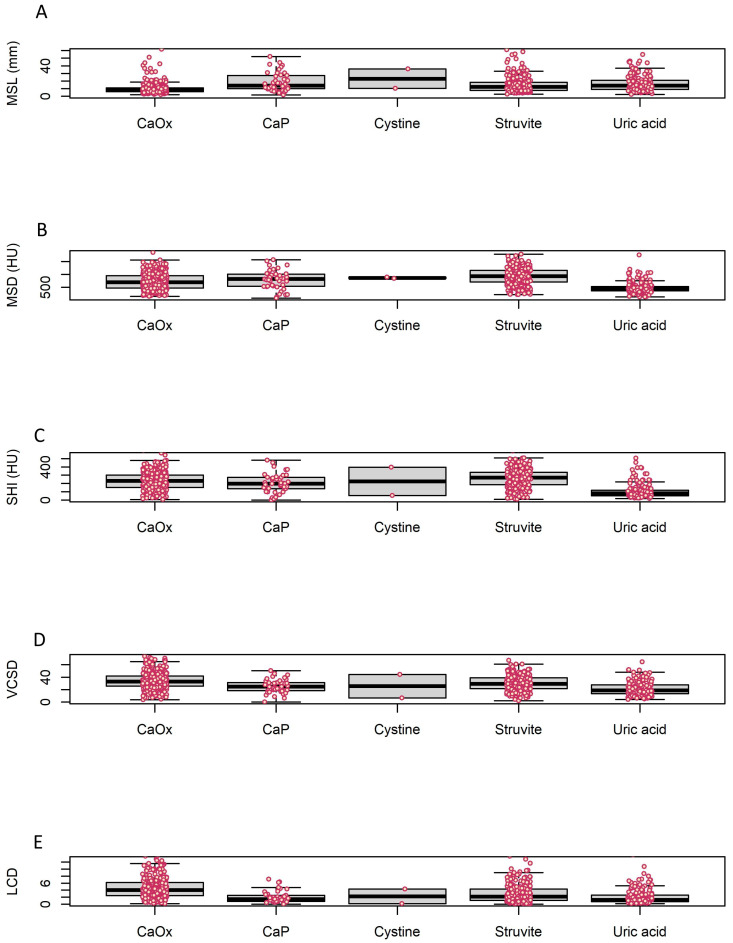
Boxplot of stone characteristics. Each box represents the interquartile range (IQR), and the upper and lower bars represent Q1 − 1.5×IQR and Q3 + 1.5×IQR. (**A**) Maximal stone length (MSL), (**B**) Mean stone density (MSD), (**C**) Stone heterogeneity index (SHI), the (**D**) Variation coefficient of stone density (VCSD), and (**E**) Linear calculus density (LCD) from non-contrast computed tomography. CaOx: calcium oxalate.

The mean age of the 790 patients was 56.4 ± 15.6 years. The mean values of the determinants were as follows: MSL, 15.4 ± 47.4 mm; MSD, 735.5 ± 347.4 HU; SHI, 213.0 ± 123.6 HU; VCSD, 29.5 ± 13.6; LCD, 3.5 ± 2.9; and urine pH, 6.0 ± 0.9. All patients were classified according to the Mayo Clinic system, and 335 patients (42.4%) were in the CaOx group. The MSL of the CaOx group was comparatively shorter than other groups; however, there was no statistically significant difference (9.8 ± 7.0, *p* = 0.052). There were significant differences among the stratified groups in all the NCCT parameters (*p* < 0.001), as shown in Table 1 and Figure 1.

### 3.2. Predictive Model for CaOx Stone

After confirming the characteristics of each stone component, all of the enrolled patients were divided into CaOx and non-CaOx groups to find a predictive model for CaOx stones. Compared to non-CaOx stones, CaOx stones were found significantly more often at younger ages (54.5 ± 15.5 years vs. 57.8 ± 15.6 years; *p* = 0.004); however, there was no difference between sexes (64.2% vs. 61.3%). As shown in Table 2, the MSL of the CaOx group was smaller than that of the non-CaOx group (9.8 ± 7.0 mm vs. 19.5 ± 61.8 mm; *p* = 0.001), and among the NCCT parameters, the SHI (230.9 ± 107.5 HU vs. 199.8 ± 132.8 HU; *p* < 0.001), VCSD (33.8 ± 13.5 vs. 26.3 ± 12.7; *p* < 0.001), and LCD (4.7 ± 3.1 vs. 2.6 ± 2.3; *p* < 0.001) of the CaOx group were higher than those of the non-CaOx group.

However, there was no difference in the MSD between the two groups (722.6 ± 324.1 HU vs. 744.9 ± 363.7 HU; *p* = 0.365). In a univariate logistic regression model, there were significant differences in the MSL, SHI, VCSD, and LCD between the two groups (*p* < 0.001) (Table 3). In multivariate analyses, lower MSL and higher SHI, VCSD, and LCD were independent predictors for CaOx stones (*p* < 0.001).

**Figure 2:**
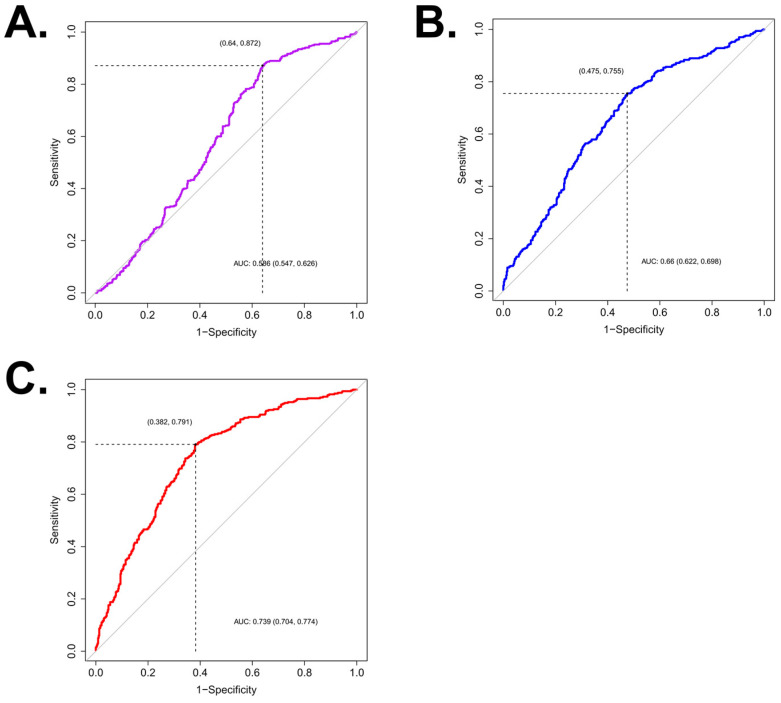
Receiver operating characteristic (ROC) curve of calcium oxalate stones for (**A**) stone heterogeneity index (SHI), AUC = 0.586 (95% CI: 0.547–0.626), cut-point of 110.60 HU; (**B**) variation coefficient of stone density (VCSD), AUC = 0.66 (95% CI: 0.622–0.698), cut-point of 25.68; (**C**) linear calculus density (LCD), AUC = 0.739 (95% CI: 0.704–0.774), cut-point of 2.25. AUC, the area under the ROC curve.

The area under the ROC curve (AUC) of SHI was 0.586 (95% CI: 0.547–0.626), with a cut-point of 110.60 HU, a sensitivity of 0.872 (95% CI: 0.831–0.906), a specificity of 0.361 (95% CI: 0.316–0.406), a positive predictive value of 0.501 (95% CI: 0.452–0.586), and a negative predictive value of 0.792 (95% CI: 0.734–0.823) for predicting CaOx stones (Figure 2A). The AUC of VCSD was 0.66 (95% CI: 0.622–0.698), with a cut-point of 25.68, a sensitivity of 0.755 (95% CI: 0.706–0.800), a specificity of 0.525 (95% CI: 0.478–0.572), a positive predictive value of 0.539 (95% CI: 0.492–0.603), and a negative predictive value of 0.745 (95% CI: 0.694–0.779) for predicting CaOx stones (Figure 2B).

The AUC of LCD was 0.739 (95% CI: 0.704–0.774), with a cut-point of 2.25, a sensitivity of 0.791 (95% CI: 0.744–0.833), a specificity of 0.618 (95% CI: 0.571–0.662), a positive predictive value of 0.604 (95% CI: 0.557–0.668), and a negative predictive value of 0.801 (95% CI: 0.755–0.830) in predicting CaOx stones (Figure 2C). DeLong’s test for two correlated ROC curves was performed to compare the capacity to predict CaOx stones, and LCD was superior to the SHI (*p* < 0.001), and VCSD (*p* < 0.001).

## 4. Discussion

NCCT imaging is currently the most sensitive (97%) and specific (95%) imaging modality to diagnose urinary tract stone disease [13]. Although there have been concerns regarding radiation hazards, this danger can be decreased by following a low-dose CT protocol. With the introduction of iterative reconstruction technology, stone protocol CT scans can be performed at lower mA and lower kVp, permitting a substantial radiation dose reduction. In their meta-analysis study, Xiang et al. have shown that low-dose CT diagnosed urinary stones with a sensitivity of 93.1% and a specificity of 96.6% [14].

Traditionally, patient history, urine pH, urinary crystals, urease-positive organisms, and plain radiographs were employed to predict stone composition. In addition to the differential diagnosis of urolithiasis, some researchers have pointed out that CT attenuation differs according to the composition of the urinary stone. In 1981, Federle et al. reported varying calculi compositions based on characteristic CT-attenuation values [15]. As SWL has become an effective and non-invasive treatment option, urologists have tried to identify patients who would be better served by SWL, which has a relatively low success rate as a single treatment (46% to 56%) [16]. Joseph et al. have assessed the correlation between the mean attenuation value of renal calculus with the SWL treatment outcome [17]. Many stones encountered in the clinical setting do not consist of a single component and have various structural differences, which are expressed as stone heterogeneity. Zarse et al. have pointed out that the internal stone structure correlates with the fragility of the calcium oxalate monohydrate (COM) calculi [18]. From this perspective, studies on factors affecting heterogeneity have been published sequentially. Lee et al. have suggested the standard deviation of urinary stone attenuation (SHI) as an indicator of heterogeneity and showed correlations between the SHI and the SWL success rates [7]. VCSD, proposed by Yamashita et al., is a stone heterogeneity indicator ensuring that the standard deviation is associated with the mean value [8].

Within these parameters, some reports have attempted to elucidate the correlations between the stone compositions. Lee et al. have shown that the MSD and SHI were lower in uric acid stones than in other stone types [9]. Kim et al. have compared various NCCT parameters and urinary pH to predict uric acid stones and suggested SHI as the most effective predictor of uric acid stones with a cut-point of 140.4 HU [10].

Although differing by region, CaOx is the most frequently reported urinary stone component (46–80%) [19,20]. Two different hydrated forms of CaOx are whewellite (COM) and weddellite (calcium oxalate dihydrate; COD). The incidence of COM is higher than that of COD, and due to its hardness, fragmentation by SWL is complex compared to the other types of stones [21]. The recurrence of CaOx is dependent on several metabolic factors, including hypercalciuria, hyperoxaluria, and hyperuricosuria [22]. A 24-h urine sample should be collected to diagnose recurrent CaOx stone formation. However, a previous report indicates that a 24-h urine sample alone does not accurately predict the stone type and composition [23].

Dual-energy computed tomography (DECT) is a technology that may improve our ability to determine stone composition. Dual-energy scanning involves simultaneous scanning using two different energies, permitting tissue characterization to help differentiate stone composition. Ahn et al. have suggested that DECT can be an effective method to identify CaOx and UA stones [24]. However, the higher radiation hazard and the higher price compared to low-dose, single-energy NCCT are obstacles to widespread use. Therefore, this study focused on the NCCT parameters and tried to figure out which variable can effectively predict CaOx stones to provide an efficient treatment modality.

Motley et al. proposed the HU density, which is the HU value of the stone divided by the largest transverse diameter in millimeters of each stone, and showed that the HU density better-characterized differences in radiodensities among urinary stones compared with the HU value alone [25]. In their study, Zarse et al. have discovered that COM stones with homogeneous structure in the CT require almost twice the shock waves to fragment than similar compositional stones with heterogeneous structural features and suggested that the stone morphology, rather than the X-ray attenuation, correlates with fragility to SWL. Stones with internal homogeneity have a uniform internal structure, are more rigid, and are difficult to break with lithotripsy. On the other hand, stones with internal heterogeneity have areas of low attenuation or internal voids within the stone component. The internal heterogeneity is an indication of high stone fragility, and the internal irregularities within-stone structures facilitate the easy disintegration of stones on SWL. This result is consistent with the findings of Lee et al., that the higher the SHI, the better the SWL treatment outcome. In other words, SHI, as well as MSD, may be utilized to discriminate the components of urolithiasis [7].

Yamashita et al. have proposed a variation coefficient (the standard deviation divided by the mean value), which is generally used to compare dispersion between multiple groups with different average values. They showed that VCSD is a better predictor of SWL success than MSD and SHI [8]. Taking these points together, we proposed LCD by putting the heterogeneity of the stone and diameter into one equation. In the current study, the mean MSL of all stone types was 15.4 ± 47.4 mm, and that of CaOx stones was 9.8 ± 7.0 mm, which is relatively shorter, but without statistical significance (*p* = 0.052). The LCD value, inversely proportional to the stone diameter, was higher in CaOx than the average LCD of all stone types (4.7 ± 3.1 vs. 3.5 ± 2.9, *p* < 0.001). After dividing the stones into the CaOx and non-CaOx groups, the AUC values of each NCCT parameter were obtained, and it was confirmed that LCD is a model with higher explanatory power (AUC 0.739 with a cut-point of 2.25) compared to other parameters.

There are several points to consider about the results of this study. First, most kidney stones that form in adults contain a majority of CaOx; LCD, with a sensitivity of 0.791 and a specificity of 0.618, may be weak in diagnosing CaOx stones. These results may be influenced by the classification system used in this study. As mentioned earlier, we classified the stone analysis result according to the Mayo Clinic classification system. Lieske et al. stated in their article that they examined the distribution of stone types and the effects of demographics and calendar month (season) on stone composition [12]. As such, Mayo Clinic’s research is focused on epidemiological factors, and some stone groups, including the struvite and uric acid groups, are classified regardless of the percentage of the major component, so the characteristics of each mineral component on the CT image might not be accurately reflected.

From the previous study by Kim et al., the CaOx group was 271/420 (56%), of which pure COM was 101/271 (37%) [10]. In addition, from a large Korean database study classified with the Mayo Clinic classification system, the CaOx group was 15,228/32,807 (46.4%), of which pure COM was 32.3% in total [20]. In our study, 335 of 790 were classified as CaOx, and pure COM was 28% in total. Therefore, even though CaOx accounts for 70–80% of the total composition of stones generally, there are many mixed types in a real clinical setting, so the sensitivity of 0.791 confirmed in our study using the Mayo classification can be clinically meaningful to evaluate a newly developed parameter.

Since stone analysis analyzes only a part, not the whole of the actual stone, there is a possibility of sampling error, and this point acts as a limitation in stone analysis research. Regarding CT image characteristics from our cases, according to the HU values for each group presented in Figure 1, the stones that had an HU value above 1000 are included in the uric acid group. In our study, among 178 cases classified as UA group, 162 cases were pure UA stone and 16 cases (8.9%) with other components exceeding 50%. In particular, there were three cases with UA 100% with MSD exceeding 1000, and two of them had relatively large MSL of 18 mm and 21 mm. Therefore, it is likely that they were non-representative of the larger stone burden. However, one of them had an LCD value of 4.46, and according to the conclusion of this study, it is highly likely to be a calcium oxalate stone. Considering these points, it is suggested that LCD can help overcome the sampling error of stone analysis.

We divided the patients into two groups, the CaOx, and the non-CaOx groups. This classification might seem inappropriate considering that the CaOx group includes COD, which is relatively easy to fragment, and brushite (CaP) and cystine stones, resistant to SWL, are in the non-CaOx group. In our study, among 335 stones classified as CaOx group, 9 cases did not contain COM at all, and 16 cases with a higher proportion of COD than COM were identified in a total of 25 stones (7.4%). Moreover, the number of stones in the CaOx group containing 60% or more of COM was 282/335 (84%), and the proportion of brushite and cystine that are resistant to ESWL in the non-CaOx group was relatively low (9.6%). Therefore, this classification can be clinically applicable in discriminating CaOx stones.

Next, the MSL values for the non-CaOx group seem divergent, with a standard deviation of over 60 mm. In this study, we included cases where percutaneous nephrolithotomy (PCNL) or endoscopic-combined intrarenal surgery (ECIRS) that was commonly selected for large stones was done. In our study, the MSL of the CaOx group is relatively low, as in Table 1. Previous studies on large stone burdens, such as the staghorn stone, showed a relatively low proportion of CaOx composition compared to that in total urolithiasis (7–24%) [26,27,28,29,30]. We attributed these differences to the pathophysiologic formation process of each stone composition. Many researchers are attempting to elucidate the mechanism of CaOx renal stone formation. It has been found that various causes are involved, and renal tubular cell injury is regarded as a major risk factor for the initial formation of urinary stones, which is triggered by reactive oxygen species and oxidative stress. Some researchers have suggested that CaOx kidney stones form while attached to Randall plaques, the subepithelial deposits on renal papillary surfaces. Meanwhile, in the case of the struvite stone, “struvite-apatite dust” is formed around the bacteria and facilitates crystal growth. Crystallization may occur both intra- and peri bacterially. Stone propagation occurs extremely quickly because of the constant supply of reactants and the alkaline milieu, in which struvite and apatite are poorly soluble [31,32,33,34,35,36]. To our knowledge, the size of a CaOx stone has not been regarded as important. When combined with HU parameters, it can be an important factor in predicting CaOx stones. Therefore, the LCD value that divides the HU parameter by MSL can be more distinct in relatively small CaOx stones. Moreover, considering that the HU value tends to increase when the size of the stone is large [37], the LCD that divides the HU parameter by MSL can be useful in discriminating CaOx stone. In addition, since CaOx stones have more than 50% recurrence [38] and need metabolic work-up, predicting CaOx stones in advance is needed to set up such a follow-up plan.

An additional point to consider is the HU value measured from the CT image. As previous studies revealed, various factors affect the measurement of CT-attenuation values of calculus, mainly beam collimation width, stone size, and X-ray energy levels. HU values decrease when the size of the stone is smaller and collimation is wider. Saw et al. reported that using the 1-mm collimation data, differentiating stone components with as little overlap as possible, but with increasing collimation width, the ability to differentiate stone compositions was lost, and the attenuation values were consistently lower at larger collimation widths. At 10-mm collimation, some uric acid stones with a diameter less than 6 mm and other stones with a diameter less than 4 mm could go undetected because of very low attenuation and partial volume effects [37]. Regarding the X-ray energy level, Tublin et al. reported that the conspicuity of small calculi at CT increases with higher kVp and mA settings, with higher kilovolts being particularly important [39]. However, Bellin et al. reported contradictory results that CT-attenuation values decreased as X-ray energy levels increased, comparing HU values calculated at 80, 120, and 140 kV [40]. Meanwhile, Zarse et al. pointed out that bone windows/level settings should be used for localizing the regions of interest to show minor stone components clearly, and considering that point, the HU value data in this study were obtained under bone window setting [41].

In addition, the following limitations exist in measuring the CT-attenuation value. Measuring stone density through CT can cause interobserver variability, and there may be a certain variability of the CT numbers obtained from scanning with different CT scanners manufactured by different companies and even among different scanners of the same manufacturer and model. Therefore, more discussions on the standardization of measurement are needed to evaluate CT parameters and utilize them.

A single-center and retrospective study has a potential selection bias. However, we collected a relatively large patient population, including those who underwent SWL and endoluminal stone surgeries, including percutaneous nephrolithotomy and ureteroscopic surgeries. In the current study, all data were collected from a single tertiary medical center. For this reason, most analyzed stones were collected through surgical treatment (3.16% from spontaneous passing and 1.39% from SWL). This may act as a type of selection bias; however, stones treated with SWL are challenging to obtain and analyze, and stones that can be treated with SWL are relatively small and radiopaque. Considering the situation, if the SWL data are included, stones by SWL can likely be included in the CaOx group, which could increase the value of the study.

## 5. Conclusions

Among the NCCT parameters, the SHI, VCSD, and LCD all showed statistical differences between the CaOx and the non-CaOx groups, but LCD had the highest predictive value for CaOx with a cut-point of 2.25. Through this study, it was found that LCD can act as an indicator to predict CaOx stone. However, further studies will be needed to increase the predictability of this new parameter and apply it for clinical usage.

## Figures and Tables

**Table 1:** Summary of patients and urinary tract stone characteristics according to the composition of calculi.

	Total (n = 790)	Struvite (n = 239)	Cystine (n = 2)	Uric Acid (n = 172)	CaOx (n = 335)	CaP (n = 42)	*p*-Value
Age	56.4 ± 15.6	55.7 ± 15.0	42.5 ± 30.4	62.2 ± 13.9	54.5 ± 15.5	52.2 ± 20.8	<0.001 ^a^
Sex							
Male	494 (62.5%)	129 (54.0%)	1 (50.0%)	130 (75.6%)	215 (64.2%)	19 (45.2%)	<0.001 ^b^
Female	296 (37.5%)	110 (46.0%)	1 (50.0%)	42 (24.4%)	120 (35.8%)	23 (54.8%)	
MSL	15.4 ± 47.4	21.8 ± 84.7	23.1 ± 18.0	16.5 ± 10.1	9.8 ± 7.0	18.6 ± 12.3	0.052 ^a^
MSD	735.5 ± 347.4	919.9 ± 339.0	864.8 ± 38.3	488.8 ± 224.3	722.6 ± 324.1	792.3 ± 362.0	<0.001 ^a^
SHI	213.0 ± 123.6	263.0 ± 116.5	225.1 ± 242.9	109.1 ± 95.6	230.9 ± 107.5	210.8 ± 137.3	<0.001 ^a^
VCSD	29.5 ± 13.6	30.0 ± 12.8	25.4 ± 27.0	21.5 ± 11.2	33.8 ± 13.5	25.5 ± 11.2	<0.001 ^a^
LCD	3.5 ± 2.9	3.0 ± 2.5	2.2 ± 2.9	2.1 ± 2.1	4.7 ± 3.1	2.0 ± 1.7	<0.001 ^a^
Urine pH	6.0 ± 0.9	6.3 ± 0.9	5.8 ± 1.1	5.4 ± 0.6	6.0 ± 0.8	6.8 ± 1.0	<0.001 ^a^

Data expressed as mean ± standard deviation or number. CaOx: calcium oxalate, CaP: calcium phosphate, MSL: maximal stone length (mm), MSD: mean stone density (HU), SHI: stone heterogeneity index (HU), VCSD: variant coefficient of stone density, LCD: linear calculus density. ^a^ One-way ANOVA. ^b^ Pearson’s chi-squared tests with Yates’ continuity correction.

**Table 2:** Summary of patient and urinary tract stone characteristics between CaOx group and non-CaOx group.

	Total (N = 790)	CaOx (N = 335)	Non-CaOx (N = 455)	*p*-Value ^a^
Age	56.4 ± 15.6	54.5 ± 15.5	57.8 ± 15.6	0.004
Sex				0.455
Male	494 (62.5%)	215 (64.2%)	279 (61.3%)	
Female	296 (37.5%)	120 (35.8%)	176 (38.7%)	
MSL	15.4 ± 47.4	9.8 ± 7.0	19.5 ± 61.8	0.001
MSD	735.5 ± 347.4	722.6 ± 324.1	744.9 ± 363.7	0.365
SHI	213.0 ± 123.6	230.9 ± 107.5	199.8 ± 132.8	<0.001
VCSD	29.5 ± 13.6	33.8 ± 13.5	26.3 ± 12.7	<0.001
LCD	3.5 ± 2.9	4.7 ± 3.1	2.6 ± 2.3	<0.001
Urine pH	6.0 ± 0.9	6.0 ± 0.8	6.0 ± 0.9	0.382

MSL = maximal stone length (mm), MSD = mean stone density (HU), SHI = stone heterogeneity index (HU), VCSD = variation coefficient of stone density (SHI/MSD × 100), LCD = linear calculus density (VCSD/MSL). ^a^ Pearson’s chi-squared tests with Yates’ continuity correction.

**Table 3:** Logistic regression models for predicting calcium oxalate stones shown by test parameters.

	Odds Ratio	95% CI	*p*-Value
Univariate			
Age	0.987	0.978–0.996	0.004
Sex	1.130	0.844–1.516	0.412
MSL	0.906	0.884–0.927	<0.001
MSD	0.999	0.999–1.000	0.373
SHI	1.002	1.001–1.003	<0.001
VCSD	1.044	1.032–1.056	<0.001
LCD	1.359	1.277–1.450	<0.001
Urine pH	1.072	0.914–1.258	0.392
Multivariate (with MSL & SHI)			
Age	0.989	0.979–0.999	0.028
MSL	0.904	0.881–0.926	<0.001
SHI	1.002	1.001–1.004	<0.001
Multivariate (with MSL & VCSD)			
Age	0.991	0.981–1.001	0.080
MSL	0.923	0.900–0.944	<0.001
VCSD	1.028	1.016–1.041	<0.001
Multivariate (with LCD)			
Age	0.995	0.985–1.005	0.296
LCD	1.352	1.270–1.444	<0.001

MSL; maximal stone length (mm), MSD; mean stone density (HU), SHI; stone heterogeneity index (HU), VCSD; variant coefficient of stone density, LCD; linear calculus density.

## Data Availability

Data are available upon request to the corresponding author.
